# A multicenter clinical study to determine the efficacy of a novel fenugreek seed (*Trigonella foenum-graecum*) extract (Fenfuro™) in patients with type 2 diabetes

**DOI:** 10.3402/fnr.v60.32382

**Published:** 2016-10-11

**Authors:** Narsingh Verma, Kauser Usman, Naresh Patel, Arvind Jain, Sudhir Dhakre, Anand Swaroop, Manashi Bagchi, Pawan Kumar, Harry G. Preuss, Debasis Bagchi

**Affiliations:** 1 Department of Physiology, King George's Medical University, Lucknow, India; 2 Department of Medicine, King George's Medical University, Lucknow, India; 3 Cepham Research Center, Piscataway, NJ, USA; 4 Research & Development, Chemical Resources, Panchkula, Haryana, India; 5 Department of Biochemistry, Georgetown University Medical Center, Washington, DC, USA; 6 Department of Medicine, Georgetown University Medical Center, Washington, DC, USA; 7 Department of Pathology, Georgetown University Medical Center, Washington, DC, USA; 8 Department of Pharmacological and Pharmaceutical Sciences, College of Pharmacy, University of Houston, Houston, TX, USA

**Keywords:** fenugreek seed extract (Fenfuro), type 2 diabetes (T2D), blood pressure, blood sugar, C-peptide, HbA1c, safety

## Abstract

**Background:**

*Trigonella foenum-graecum* (fenugreek) seeds are known to exhibit potent antioxidant, hypoglycemic, and nephroprotective activities, as well as serve as excellent membrane stabilizers especially because of their content of novel furostanolic saponins. Our previous studies exhibited the broad spectrum safety and efficacy of Fenfuro, a novel *T. foenum-graecum* seed extract enriched in furostanolic saponins, in type 2 diabetes (T2D) in rats.

**Design:**

This multicenter, randomized, placebo-controlled, double-blind, add-on clinical study evaluated over a period of 90 consecutive days the efficacy of Fenfuro (daily dosage: 500 mg bid) in 154 subjects (male: 108; female: 46; age: 25–60 years) with T2D.

**Methods:**

This study examined the body weight, blood pressure, and pulse rate, as well as the efficacy of Fenfuro on fasting and post-prandial plasma sugar (mg/dL), glycosylated hemoglobin (HbA1c), and fasting and post-prandial C-peptide levels.

**Results:**

Fenfuro caused significant reduction in both fasting plasma and post-prandial blood sugar levels. Approximately 83% of the subjects reported decreases in fasting plasma sugar levels in the Fenfuro-treated group as compared to 62% in the placebo group, while 89% of the subjects demonstrated reduction in post-prandial plasma sugar levels in the Fenfuro-treated group as compared to 72% in the placebo group. HbA1c levels were reduced in both placebo and treatment groups. The decrease in HbA1c levels was significant in both groups as compared to respective baseline values. A significant increase in fasting and post-prandial C-peptide levels compared to the respective baseline values was observed, while no significant changes in fasting and post-prandial C-peptide levels were observed between the two groups. No significant adverse effects were observed by blood chemistry analyses. Furthermore, 48.8% of the subjects reported reduced dosage of anti-diabetic therapy in the Fenfuro-treated group, whereas 18.05% reported reduced dosage of anti-diabetic therapy in the placebo group.

**Conclusion:**

In summary, Fenfuro proved safe and efficacious in ameliorating the symptoms of T2D in humans.

Diabetes mellitus is a group of polygenic disorders in which a person has high blood glucose, where insulin production is inadequate, and/or the body's cells do not respond properly to insulin. Diabetes is now a challenge to health professionals (1–5). Patients with high blood sugar will experience polyuria, polydipsia, and polyphagia (1, 2). Recent statistics by the International Diabetes Federation in 2014 state that there are 387 million people living worldwide with diabetes and its complications. However, 46.3% of the afflicted people remain undiagnosed, and it is expected that this will increase in an alarming rate to 205 million by 2035 (3). Some of the potential complications of diabetes include cardiovascular complications; macrovascular and microvascular complications including neuropathy, nephropathy, retinopathy, and blindness; foot damage; hearing impairment; various skin diseases; and Alzheimer's disease (1, 2, 5). Atherosclerosis is also a major macrovascular complication of diabetes. Unfortunately, it is the leading cause of morbidity and mortality in this modern era, resulting in stroke and peripheral circulatory disorders (5).

The pathogenesis of diabetes involves both genetic and environmental factors that adversely affect insulin secretion and regulation (5). Genomics studies have attempted to identify genetic variants that contribute to the development of diabetes (6). Studies suggest that epigenetic phenomena may play a major role in the development of diabetes (7).

Presently, five classes of drugs are currently used to regulate blood glucose (8, 9). In the geriatric population, sulfonylureas and metformin, a biguanide, are the most widely used oral anti-diabetic agents (8, 10). Meglitinides, thiazolidinediones, alpha-glucosidase inhibitors, and incretins are additional drugs used to regulate blood glucose (8–11). Unfortunately, these drugs often demonstrate adverse side effects (1, 8).

Healthy lifestyle including proper nutrition, in conjunction with regular exercise and physical activity, has been demonstrated to modulate the severity of type 2 diabetes (T2D) (1). A significant number of medicinal plants including *Allium cepa*, *Allium sativum*, *Aloe vera*, *Brassica juncea*, *Cajanus cajan*, *Coccinia indica*, *Caesalpinia bonducella*, *Curcuma longa*, *Eugenia jambolana*, *Ficus benghalensis*, *Gymnema sylvestre*, *Momordica charantia* L., *Mucuna pruriens*, *Murraya koenigii*, *Ocimum sanctum*, *Pterocarpus marsupium Roxb*., *Swertia chirayita*, *Syzygium cumini*, *Tinospora cordifolia*, *and Trigonella foenum-graecum* L. have demonstrated varying degree of hypoglycemic and anti-hyperglycemic activity in experimental and clinical anti-diabetic models (1, 12–18). Many phytochemicals including alkaloids, flavonoids, phenolics, and terpenoids have displayed significant anti-diabetic potential. Particularly, schulzeines A, B, and C, radicamines A and B, 2,5-imino-1,2,5-trideoxy-L-glucitol, beta-homofuconojirimycin, myrciacitrin IV, dehydrotrametenolic acid, corosolic acid, 4-(alpha-rhamnopyranosyl)ellagic acid, and 1,2,3,4,6-pentagalloylglucose have shown significant anti-diabetic activities (1, 12–18). A database for anti-diabetic plants with clinical/experimental trials has already been established (17, 18). A significant number of studies demonstrate the antioxidant and hypoglycemic efficacy of fenugreek seeds (*T. foenum-graecum*) (19–26); however, a more recent study reported nephroprotection of fenugreek seeds against alcohol-induced intoxication in rats (27). This study also demonstrated, using transmission electron microscopy and ultrastructural analysis (27), that fenugreek seeds prevent cellular deterioration. Finally, fenugreek seeds improved renal morphology and function (27).

Our earlier animal studies revealed the broad-spectrum safety and anti-diabetic efficacy of fenugreek seed extract (28, 29). In the present study, we assessed the efficacy of a novel, patented fenugreek seed extract enriched in furostanolic saponins (Fenfuro), to determine its anti-diabetic efficacy in a multicenter, placebo-controlled, double-blind, add-on clinical study in 154 diabetic subjects (male: 108; female: 46; age: 25–60 years) over a period of 90 consecutive days.

## Materials and methods

### Novel Trigonella foenum-graecum seed extract (Fenfuro)

A patented *T. foenum-graecum* seed extract (Fenfuro™, Batch No F0413, Mfg Date April 2013, US Patent 8,754,205B2 17 June 2014; US008217165B2 10 July 2012, Cepham Inc., Piscataway, NJ, USA) (30, 31) enriched in approximately 40% furostanolic saponins was used in this study. A patent-pending water–ethanol extraction process was used to manufacture Fenfuro in a GMP-NSF certified manufacturing plant.

### Study design

This multicenter, placebo-controlled, double-blind, add-on clinical study entitled ‘Clinical evaluation of fenugreek seed extract in patients with T2D: an add-on study’ (Protocol #CR002/02/13), approved by Institutional Review Board and Institute Ethics Committee (Approval #CEC/2013/06/22/I dated 22 June 2013), was conducted in King George's Medical University (Lucknow, UP, India) and in Dr. Arvind Jain's Clinic (Agra, Uttar Pradesh, India). The study was duly approved by Institutional Ethics Committee of King George's Medical University, Uttar Pradesh, and Independent Ethics Committee, ‘Conscience Ethics Committee’, Agra, Uttar Pradesh, India. The protocol was performed in compliance and accordance with International Council on Harmonization (ICH) Guidelines for Good Clinical Practices, including the archiving of essential documents, and as per international ethical standards, guaranteed by the Declaration of Helsinki and its subsequent amendments.

All the subjects enrolled for the study were provided a consent form along with sufficient information to make a well-informed decision about their participation in this study. This consent form was submitted with the protocol for review and approved by the institution ethics committee (IEC) for the study. The formal consent of a subject, using the IEC-approved consent form, was obtained before the subjects were included in any study procedure. The consent form was signed by the subject or a legally accepted representative, and the investigator/designated research professional obtained the consent. Patient confidentiality was strictly maintained.

### Subject recruitment

In total, 154 subjects (male=108; female=46; age: 25–60 years) were recruited following the inclusion and exclusion criteria (Tables 1 and 3), and randomized into placebo and treatment groups using a computer-generated randomization code. In the treatment group, 63.6% of the subjects were males and 36.4% of the subjects were females, whereas in the placebo group, 76.6% subjects were males and 23.4% were females. There were no significant differences in mean body weight, systolic and diastolic blood pressure, and pulse rate of the treatment group as compared to the placebo group. Both groups had similar demographic distribution and the study population consisted of subjects of either sex. Each patient was given a sealed aluminum pouch containing 60 capsules equivalent to the dose for 1 month. The treatment group received Fenfuro capsules (2 capsules of 500 mg each per day), whereas the placebo group received placebo capsules, which were prepared using di calcium phosphate.

**Table 1:** Inclusion and exclusion criteria

Inclusion criteria
Male and female subjects between 25 and 60 years of age Suffering from T2D for less than 5 years HbA1c >7.5% Fasting blood glucose not exceeding 180 mg/dL On oral anti-diabetic treatment (metformin±sulfonylurea) No change in anti-diabetic therapy for the last 1 month Patients willing to give informed consent
Exclusion criteria
Diabetes other than T2D Evidence of renal disease (serum creatinine>1.5 mg/mL) Evidence of liver disease (AST/ALT>3 times of normal) Pregnant or lactating women and subjects intending pregnancy Participation in any other clinical trial within the last 30 days History of any hemoglobinopathy that may affect determination of glycosylated hemoglobin Treatment with oral anti-diabetic agents (other than metformin or sulfonylurea) during the 12 weeks before baseline History of intolerance or hypersensitivity to sulfonylurea or metformin or fenugreek seed extract

Subjects were strictly instructed to consume a prescribed vegetarian or non-vegetarian diet of approximately 2,000 kcal/day (protein 18–22%, carbohydrate 52–56%, and fat 22–26%) throughout the study period. Hence, the vegetarian or non-vegetarian diet will have no significant effect in lowering blood glucose.

### Assay kits and equipment

Assay kits for glycosylated hemoglobin (HbA1c), serum aspartate aminotransferase (AST), alanine aminotransferase (ALT), alkaline phosphatase (ALP), bilirubin, blood urea nitrogen (BUN), creatinine, fast and postprandial blood glucose, fasting and postprandial C-peptide levels, and total leukocyte count (TLC) (× 10^3^/µl) were used at baseline and at end of 90 days of treatment to demonstrate the broad-spectrum safety and efficacy of Fenfuro (Table 2). The kits were purchased from the authorized distributor of Johnson & Johnson Ltd (Asha Medical Store, Hewat Road, Lucknow, Uttar Pradesh, India).

**Table 2:** Assessment of efficacy

Time intervals	Clinical examinations
At baseline	Glycosylated hemoglobin (HbA1c)
	Liver function tests (AST, ALT, ALP and bilirubin)
	Renal function tests (urea and creatinine)
	Cardiovascular function test (creatinine)
	Hematogram
	Fasting blood glucose
	Postprandial blood glucose
	Serum C-peptide
	Serum bilirubin
	Total leukocyte count
First and second month follow-up visit	Liver function test (AST, ALT, ALP and bilirubin)
	Renal function test (urea and creatinine)
	Fasting blood glucose
	Postprandial blood glucose
	Serum bilirubin
On completion of treatment (third month)	HbA1c
	Liver function test (AST, ALT, ALP and bilirubin)
	Renal function test (urea and creatinine)
	Hematogram
	Fasting blood glucose
	Postprandial blood glucose
	Serum C-peptide
	Serum bilirubin
	Total leukocyte count

#### Assessment of safety

Clinical biochemistry evaluations including serum BUN (mg/dL), serum creatinine (mg/dL), serum bilirubin (mg/dL), serum AST (U/L), serum ALT (U/L), serum ALP activity (U/L), hemoglobin (Hb), and TLC (× 10^3^/µl) were meticulously checked at the baseline and end of 90 days treatment. Biochemical and hematological parameters were measured at 0, 30, 60, and 90 days of treatment (Table 2).

#### Assessments of efficacy

This study recruited subjects suffering from T2D for not more than 5 years, which is clearly mentioned in the inclusion and exclusion criteria. We also clearly indicated in the inclusion criteria that HbA1c in the recruitment group was>7.5%, while fasting blood glucose was not exceeding 180 mg/dL. Thus, the recruited subjects in the baseline had HbA1c>7.5% and fasting blood glucose less than 180 mg/dL. Then, these recruited subjects were divided into two groups, 1) placebo and 2) Fenfuro treatment group. Both placebo and Fenfuro-treated groups were on metformin. Hence, the effect of metformin in lowering blood glucose would be similar in both the groups.

#### Blood glucose

Fasting and postprandial plasma glucose levels (mg/dL) were measured at 0, 30, 60, and 90 days of treatment using kits from authorized distributor of Johnson & Johnson Ltd.

#### Glycosylated hemoglobin

HbA1c levels were measured at 0 and 90 days of treatment using kits manufactured by Bio-Rad Laboratories (Irvine, CA, USA).

#### C-peptide

Fasting and postprandial C-peptide levels were assessed at 0 and 90 days of treatment using the kits manufactured by DiaSorin S.p.A. (Saluggia, Italy).

#### Adverse events

Subjects were advised to record adverse events (if any) during the duration of the study. At each visit, the subjects were asked if they had experienced any uncomfortable problems or difficulties. Thus, adverse event reporting was strictly enforced.

#### Study compliance

Allocation of Fenfuro was accomplished by site staff. Distribution of the investigational product was maintained in the IP accountability log provided by the sponsor. Each entry was maintained separately with the date/signature of the principal investigator and study coordinator.

The person responsible for the distribution of the product had also signed the IP accountability log. The accountability log was readily available at the time of audit.

### Statistical analysis

Data is expressed as mean±SD. The baseline characteristics were compared with the outcome following completion of the dosing period. Appropriate parametric and non-parametric tests were used according to the data.

## Results

### Efficacy of Fenfuro on fasting and postprandial plasma sugar in the placebo- and Fenfuro-treated subjects

Fenfuro treatment caused a significant decrease in fasting plasma sugar levels as compared to the corresponding placebo-treated group. Approximately 6.69, 10.31, and 21.98% decreases in fasting plasma sugar levels were observed at 30, 60, and 90 days of Fenfuro treatment, respectively, whereas under these same conditions and time points, approximately 3.2, 1.2, and 7.6% reduction in fasting plasma sugar levels were observed in the placebo group (Table 3). A significant or more significant decrease in fasting plasma sugar was observed after 30, 60, and 90 days of Fenfuro treatment compared to the baseline. In the placebo group, no significant change in fasting plasma sugar levels was observed after 30 and 60 days of treatments as compared to baseline. However, a significant decrease in fasting plasma sugar level was observed at 90 days post-treatment as compared to its respective baseline (Table 3).

**Table 3:** Effect of Fenfuro on fasting and postprandial plasma sugar levels

Parameters	Groups	Baseline	30-days	60-days	90-days
Fasting plasma sugar levels (mg/dL)	Placebo group	152.96±26.97	148.13±37.72	151.12±48.11	141.81±39.42
	*p*-value	–	0.192, ns	0.721, ns	0.202, ns
	Treatment group	151.31±24.42	141.19±33.59	135.70±44.87	118.05±25.33
	*p*-value	–	0.015*	0.007**	0.000**
Postprandial plasma sugar levels (mg/dL)	Placebo group	250.07±75.90	231.05±73.87	226.31±77.97	206.57±72.41
	*p*-value	–	0.027*	0.012*	0.000**
	Treatment group	251.01±68.88	216.64±74.69	199.38±70.57	174.78±54.90
	*p*-value	–	0.000**	0.000**	0.000**

A postprandial glucose test is a blood glucose test that determines the amount of glucose in the blood after a meal. Data are expressed as mean±SD.

*, ** Significant reduction; ns, not significant.

Similarly, Fenfuro treatment also caused a greater significant reduction in postprandial plasma sugar levels as compared to the placebo. Approximately 13.7, 20.6, and 30.4% decreases in postprandial plasma sugar levels were observed at 30, 60, and 90 days of Fenfuro treatment, respectively, whereas under these same conditions and time points, approximately 7.6, 9.5, and 17.4% reduction in postprandial plasma sugar levels were observed in the placebo group (Table 3).

### Efficacy of Fenfuro on glycosylated hemoglobin (HbA1c) levels in the placebo and treatment groups

HbA1c levels were reduced in both placebo- and Fenfuro-treated groups. However, the reductions were not significant.

### Effect of Fenfuro on fasting and postprandial C-peptide levels in the placebo and treatment groups

A significant increase in fasting C-peptide levels was observed as compared to the respective baseline values; however, no significant change in fasting C-peptide levels was observed between the placebo and treatment groups. Similarly, in the postprandial C-peptide levels, a significant increase was observed as compared to the respective baseline values, whereas no significant changes in the C-peptide levels were observed between the two groups (Table 4).

**Table 4:** Fasting and postprandial C-peptide levels (mg/dL) in placebo- and Fenfuro-treated subjects

				Statistical analyses	
				
Parameters	Treatment	Placebo group (mean±SD)	Treatment group (mean±SD)	Paired *t*- and *p*- value	Independent test *p*- and *t*-value
Fasting C-peptide levels	Baseline	3.28±2.29	2.60±1.68	*t*=4.283, *p*=0.001**	*t*=0.871, *p*=0.389, ns
	On completion	4.79±3.09	4.11±2.08	*t*=4.423, *p*=0.000**	*t*=1.140, *p*=0.260, ns
Postprandial C-peptide levels	Baseline	2.33±1.64	2.56±2.11	*t*=5.427, *p*=0.001**	*t*=1.285, *p*=0.205, ns
	On completion	4.47±2.53	5.40±2.78	*t*=7.210, *p*=0.000**	*t*=0.438, *p*=0.663, ns

Data are expressed as mean±SD.

** Significant reduction; ns, not significant.

### Blood chemistry analyses

Blood chemistry parameters including serum BUN, creatinine, ALT, AST, ALP activity, bilirubin, and TLC were not differing in the placebo and treatment groups as demonstrated in Table 5.

**Table 5:** Serum BUN, creatinine, ALT, AST, ALP, bilirubin and TLC levels

Parameters	Treatment	Placebo (mean±SD)	Treatment (mean±SD)	Statistical analyses
BUN (mg/dL)	Baseline	25.79±7.15	26.16±8.62	*t*=0.274, *p*=0.784, ns
	On completion	25.24±8.05	24.45±5.89	*t*=0.651, *p*=0.516, ns
Creatinine (mg/dL)	Baseline	0.80±0.17	0.83±0.26	*t*=0.888, *p*=0.376, ns
	On completion	0.79±0.18	0.75±0.17	*t*=1.389, *p*=0.167, ns
ALT (U/L)	Baseline	43.41±28.42	38.31±20.65	*t*=1.195, *p*=0.234 ns
	On completion	31.96±14.82	32.23±13.52	*t*=0.108, *p*=0.914, ns
AST (U/L)	Baseline	34.59±17.48	34.09±21.79	*t*=0.149, *p*=0.882, ns
	On completion	27.03±8.04	28.76±15.62	*t*=0.830, *p*=0.408, ns
ALP (U/L)	Baseline	107.35±31.96	106.14±48.02	*t*=0.177, *p*=0.860, ns
	On completion	95.04±30.29	93.35±30.29	*t*=0.327, *p*=0.744, ns
Bilirubin (mg/dL)	Baseline	0.55±0.25	0.53±0.29	*t*=0.439, *p*=0.661, ns
	On completion	0.58±0.59	0.47±0.18	*t*=1.383, *p*=0.169, ns
Hb (%)	Baseline	13.47±1.53	13.76±1.78	*t*=1.031, *p*=0.304, ns
	On completion	13.75±1.66	14.02±1.69	*t*=0.956, *p*=0.341, ns
TLC (× 10^3^/µL)	Baseline	2962.87±4149.82	2721.77±4139.51	*t*=0.341, *p*=0.733, ns
	On completion	2718.45±3784.73	2358.19±3562.91	*t*=0.574, *p*=0.567, ns

Data are expressed as mean±SD; ns, not significant.

## Discussion

The largest producer of fenugreek seed is India, and the seeds, leaves, and whole plant are widely used both in fresh and dried forms in the domestic purpose as an herb, spice, vegetable, and salad in India, China, and Middle Eastern countries (18–20). Interestingly, fenugreek is also becoming popular in the Western world as a medicinal herb or as a spice (32, 33). Fenugreek leaves and seeds have long been used in both Ayurvedic and Chinese medicines in the treatment of diabetes (19). A large number of preclinical and clinical studies have been conducted on fenugreek seeds (1, 22–26).

Our previous broad-spectrum safety and anti-diabetic efficacy studies were conducted in rats using a novel, patented, fenugreek seed extract (Fenfuro) that is approximately 40% furostanolic saponins. Fenugreek seeds are known to contain soluble dietary fiber, protein, vitamin C, niacin, potassium, 4-hydroxyisoleucine, lysine and selected amino acids, L-tryptophan, and selected steroidal saponins including diosgenin, yamogenin, tigogenin, and neotigogenin, demonstrated to inhibit both cholesterol absorption in the intestine and cholesterol production by the liver (34, 35). Fenugreek seeds also contain a gel-like soluble fiber that has been exhibited to combine with bile acids and lowers the triglyceride (TG) and LDL levels. The amino acids present are useful as a plus in boosting insulin sensitization and glycogen synthesis. Dietary fibers and saponins that are present are specifically known to enhance hypoglycemic activity. Additionally, dietary fiber may significantly contribute to fenugreek's activity in lowering blood glucose and cholesterol (36).

Broad-spectrum therapeutic efficacy and medicinal properties of fenugreek seeds on metabolic disorders have been demonstrated in animal studies that suggest that fenugreek may also contain a constituent which stimulates insulin production or sensitization (35, 37, 38). Earlier studies in animals demonstrated that fenugreek seed extract has the potential to slow the enzymatic digestion of carbohydrates, reduce gastrointestinal absorption of glucose, and thus reduce postprandial glucose level (32, 37, 38). Fenugreek seeds have been reported to reduce serum cholesterol level and attenuate blood glucose level and improve lipid metabolism. It has also been reported that fenugreek reduces lipid level in plasma and liver leading to the improvement of insulin sensitivity in rats with metabolic disorders (36, 39).

In summary, studies on fenugreek seeds in humans and animals demonstrate significant attenuation of glucose tolerance and improvement in the glucose-induced insulin response suggesting a potential hypoglycemic activity of fenugreek seeds (39–41). Multiple human trials on fenugreek seeds also demonstrate potential efficacy in lowering total cholesterol in people with moderate atherosclerosis or insulin- or non-insulin-dependent diabetes. A human double-blind trial has demonstrated that defatted fenugreek seeds may raise the beneficial HDL cholesterol (40, 42, 43), whereas Prasanna et al. (40) have shown that two different doses of defatted fenugreek seed powder (25 or 50 g/day) significantly lower serum cholesterol after 20 days. A clinical trial on humans suffering from T2D using 15 g of powdered fenugreek seeds with meals reported a reduced rise in blood glucose after the meal. Another similar controlled trial found that taking 2.5 g of fenugreek seed twice a day for 3 months reduced blood glucose levels in people with mild T2D (37, 38). A double-blind clinical study in subjects with T2D used 1 g of fenugreek seed extract/day over a period of 2 months and found improved blood sugar and insulin function. Fenugreek seeds have also been reported to lower total and LDL cholesterol and TG levels in people with high levels (33, 40). Another randomized trial demonstrated that fenugreek seed extract (100 g/day) lowers elevated blood glucose, TG, and other lipid levels (39, 40). It is interesting to note that Sharma et al. (43) demonstrated the efficacy of fenugreek seeds in type 1 diabetes (43).

The present investigation was designed as a multicenter, randomized, placebo-controlled, double-blind clinical study as an ‘add-on therapy’. The add-on designation means, either Fenfuro (US Patent 8,754,205B2 17 Jun 2014, standardized *Trigonella foenum-graecum* seed extract enriched in approximately 40% furostanolic saponins, dose: 500 mg bid) or placebo was given in addition to standard anti-diabetic therapy (metformin) in 154 male and female subjects (male: 108; female: 46; age: 25–60 years) with T2D over a period of 3 months. Both placebo and treatment groups consumed metformin for their anti-diabetic therapy, while the treatment group consumed Fenfuro in conjunction with metformin so the anti-diabetic effect demonstrated in the present study reflects the greater efficacy of Fenfuro in lowering blood sugar level.

This research estimated fasting and postprandial plasma sugar (mg/dL), HbA1c, and fasting and postprandial C-peptide levels over a period of 3 months. Fenfuro caused significant reduction in both fasting plasma and postprandial blood sugar levels as compared to the placebo group. Interestingly 83% reported a decrease in fasting plasma sugar levels in the treatment group as compared to 62% in the placebo group, while under these same conditions, approximately 89% of the subjects demonstrated a reduction in postprandial plasma sugar levels in the treatment group as compared to 72% in the placebo group.

Reductions in the HbA1c levels were non-significant in both groups as compared to respective baseline values. In the Fenfuro-treated group, the decrease in HbA1c level was 18.36%, while in the placebo group the reduction was 16.63% of the respective baseline values at the completion of the study. A significant increase in fasting and postprandial C-peptide levels was observed as compared to the respective baseline values; however, no significant changes in fasting and postprandial C-peptide levels were observed between the two groups.

No significant adverse events were reported by the subjects and blood chemistry analyses in the placebo and treatment groups demonstrated the broad-spectrum safety of Fenfuro in conjunction with metformin.

It is highly encouraging to mention that 48.8% of the subjects reported reduced anti-diabetic therapy in the Fenfuro-treated group, whereas 18.05% of the subjects reduced anti-diabetic therapy in the placebo group.

Overall, this study exhibits that Fenfuro in conjunction with metformin (treatment group) was significantly more effective as compared to the placebo group (metformin alone). Furthermore, Fenfuro has broad-spectrum safety and greater efficacy in ameliorating the symptoms of T2D in humans. This study also opens a new avenue that Fenfuro may be used as a treatment regimen in conjunction with metformin. Further studies are in progress to establish the molecular mechanism of action.
